# The complement factor B inhibitor iptacopan for relapsed and refractory immune thrombotic thrombocytopenic purpura

**DOI:** 10.1186/s12959-026-00854-9

**Published:** 2026-03-25

**Authors:** Minran Zhou, Xiaoqing Li, Ran Wang, Hai Zhou, Yu Hou, Ping Qin, Sai Ma, Chunyan Chen

**Affiliations:** 1 Department of Hematology, Qilu Hospital of Shandong University, Jinan, Shandong China; 2 Shandong Key Laboratory of Hematological Diseases and Immune Microenvironment, Jinan, China; 3 Shandong Provincial Clinical Research Center for Hematological Diseases, Jinan, China

**Keywords:** Immune thrombotic thrombocytopenic purpura, Iptacopan, Therapy

## Abstract

**Background:**

Thrombotic thrombocytopenic purpura (TTP) is a rare and life-threatening thrombotic microangiopathy, classified as congenital TTP (cTTP) and immune TTP (iTTP). iTTP represents the most common clinical subtype, accounting for approximately 95% of cases. Primary clinical features include microangiopathic hemolytic anemia (MAHA), thrombocytopenia, neuropsychiatric symptoms, fever, and renal involvement. Reduced ADAMTS13 activity and dysregulation of the complement pathway contribute to the pathogenesis of iTTP. First-line therapy for newly diagnosed iTTP, comprising plasma exchange and corticosteroids, demonstrates reasonable efficacy. However, some patients experience disease relapse with suboptimal response to plasma exchange. These relapsed and refractory cases pose significant therapeutic challenges and are associated with an extremely poor prognosis and high mortality.

**Case presentation:**

Here, we report a case of relapsed and refractory iTTP presenting with scleral icterus, thrombocytopenia, altered mental status, fever, and renal impairment. Despite initial treatment with plasma exchange, corticosteroids, ripertamab, and intravenous immunoglobulin, the patient’s condition deteriorated rapidly. Laboratory tests revealed elevated levels of the terminal complement complex (C5b-9) and complement factor Ba in the patient. Consequently, we initiated treatment with the complement inhibitor iptacopan, combined with plasma exchange, corticosteroids, and rituximab. The hemoglobin and platelet count increased significantly by Day 5. At the 5-week follow-up, ADAMTS13 activity, hemoglobin, and platelet counts had all normalised.

**Conclusions:**

This case is a preliminary observation. With biomarker evidence supporting alternative pathway activation (e.g., elevated Ba and sC5b-9), it reports for the first time the potential association of combined therapy involving plasma exchange, corticosteroids, rituximab, and iptacopan with rapid clinical improvement in a patient with relapsed and refractory iTTP. It provides initial experience for exploring complement alternative pathway inhibition as a potential adjunctive strategy for such patients.

## Introduction

TTP arises from a profound deficiency in the plasma enzyme ADAMTS13 activity. An ADAMTS13 activity level below 10 IU/dL confirms the diagnosis [1]. In iTTP, inhibitory antibodies against ADAMTS13 (typically IgG class) are frequently detected [2]. TTP represents a life-threatening hematological emergency characterised by rapid progression and poor prognosis, mandating urgent diagnosis and intervention [3, 4]. Long-term monitoring of ADAMTS13 levels in iTTP patients is essential for the early detection of relapse [5]. Current management combines therapeutic plasma exchange (TPE) with immunosuppressive therapy (corticosteroids and rituximab) to mitigate acute inflammation and autoantibody production [4]. A subset of patients remains refractory to these therapies. Clinical relapse in patients with iTTP is defined as a decrease in platelet count to < 150 × 10^9^/L (with other causes of thrombocytopenia ruled out), with or without clinical evidence of new ischemic organ injury, after attainment of a clinical remission. A clinical relapse should always be confirmed by documentation of severe ADAMTS13 deficiency (ADAMTS13 activity < 10%). ADAMTS13 relapse was defined as a decrease in ADAMTS13 activity to < 20% after an ADAMTS13 remission [6]. Refractory TTP is defined as persistent thrombocytopenia, lack of a sustained platelet count increment or platelet count (PLT) of < 50 × 10^9^/L, and a persistently raised lactate dehydrogenase (LDH) level (> 1.5 ULN) despite five plasma exchanges and steroid treatment [7]. Relapsed and refractory iTTP poses significant therapeutic challenges, with extremely poor prognosis and high mortality rates.

In the pathogenesis of TTP, although the role of ultra-large von Willebrand factor (VWF) multimers is well established, emerging evidence suggests that excessive activation of the alternative complement pathway may contribute to the pathophysiology of acute iTTP, indicating that complement activation also results in iTTP [8–10]. Iptacopan, an oral complement factor B inhibitor, effectively and safely blocks the complement system proximally in the alternative pathway (AP). It increases hemoglobin levels, reduces fatigue, and lowers reticulocyte and bilirubin levels, potentially avoiding transfusion requirements [11]. In this specific case of relapsed and refractory iTTP, the patient presented with severe microangiopathic hemolytic anemia. Elevated levels of sC5b-9 and complement factor Ba implicated alternative pathway activation in the pathogenesis. Consequently, we administered iptacopan alongside high-dose corticosteroids, plasma exchange, and rituximab. The patient achieved an excellent clinical response with reduced early mortality risk. This therapeutic strategy offers a valuable approach for managing similar cases.

## Case description

The patient was a young male with no prior history of chronic disease. One year ago, he was admitted with two days of altered consciousness. Initial investigations revealed severely deficient ADAMTS13 activity (< 0.2 IU/dL), elevated ADAMTS13 inhibitor (anti-ADAMTS13 IgG: 66.1 U/mL). Microangiopathic hemolysis was evident on peripheral blood smear (3% schistocytes), with a reticulocyte count of 0.33 × 10^9^/L, LDH 1014 IU/L, total bilirubin (TBIL) 65 µmol/L, and indirect bilirubin (IBIL) 56 µmol/L, along with moderate anemia and PLT 15 × 10^9^/L. Other causes were excluded (Coombs test negative; rheumatism series and lupus anticoagulants normal). Based on characteristic clinical presentation, signs, and laboratory findings, a definitive diagnosis of iTTP was established. Treatment in our institution included methylprednisolone (80 mg daily for 16 days), three sessions of therapeutic plasma exchange, intravenous immunoglobulin, and fresh frozen plasma transfusions. By discharge, the patient had achieved hematological recovery, with a platelet count of 507 × 10^9^/L and hemoglobin (Hb) of 104 g/L, along with only occasional schistocytes, indicating clinical stability.

Approximately five months ago, the patient was admitted to another hospital with disorganized speech, widespread petechiae, and ecchymoses. Laboratory tests revealed microangiopathic hemolytic anemia (9% schistocytes, Hb 43 g/L, reticulocytosis 10.73%, TBIL 60.9 µmol/L, IBIL 32.7 µmol/L), severe thrombocytopenia (15 × 10^9^/L), and profoundly deficient ADAMTS13 activity (1.51%) with positive ADAMTS13 inhibitory antibodies. Given the presence of jaundice, severe anemia, elevated reticulocyte count, and elevated bilirubin, microangiopathic hemolytic anemia was suspected. In conjunction with thrombocytopenia, cutaneous hemorrhagic manifestations, and neurological symptoms, a diagnosis of relapsed iTTP was established. The patient underwent nine sessions of plasma exchange at the referring hospital, in combination with corticosteroids, ripertamab, and intravenous immunoglobulin. However, due to persistent agitation and psychiatric disturbances suggesting suboptimal therapeutic response, the patient was transferred to our hospital, diagnosed as relapsed and refractory iTTP.

Emergency investigations in our institution revealed microangiopathic hemolytic anemia (3% schistocytes, Hb 45 g/L, TBIL 68 µmol/L, IBIL 48 µmol/L), severe thrombocytopenia (19 × 10^9^/L), and a negative Coombs test. Given the patient’s history and the classic triad of microangiopathic hemolysis, thrombocytopenia, and neurological symptoms, a diagnosis of relapsed TTP was established. Initial emergency management included plasma exchange (2600 mL total), methylprednisolone (40 mg once daily), red blood cell transfusions (4 units total), and antibiotics. However, after seven days, the patient exhibited disease progression with worsening confusion, agitation, extensive ecchymoses, bilateral lower lobe dependent atelectasis on chest CT, and minor hemorrhagic foci on cranial CT, necessitating transfer to the emergency intensive care unit (EICU). Intensive care treatment comprised further plasma exchange (1500 mL), high-dose methylprednisolone (120 mg once daily), additional red cell transfusions (4.5 units), antibiotics, antipyretics, and sedation. Despite nine days of therapy, laboratory results showed persistent severe anemia (Hb 49 g/L) and worsening thrombocytopenia (PLT 7 × 10^9^/L).

**Fig. 1:**
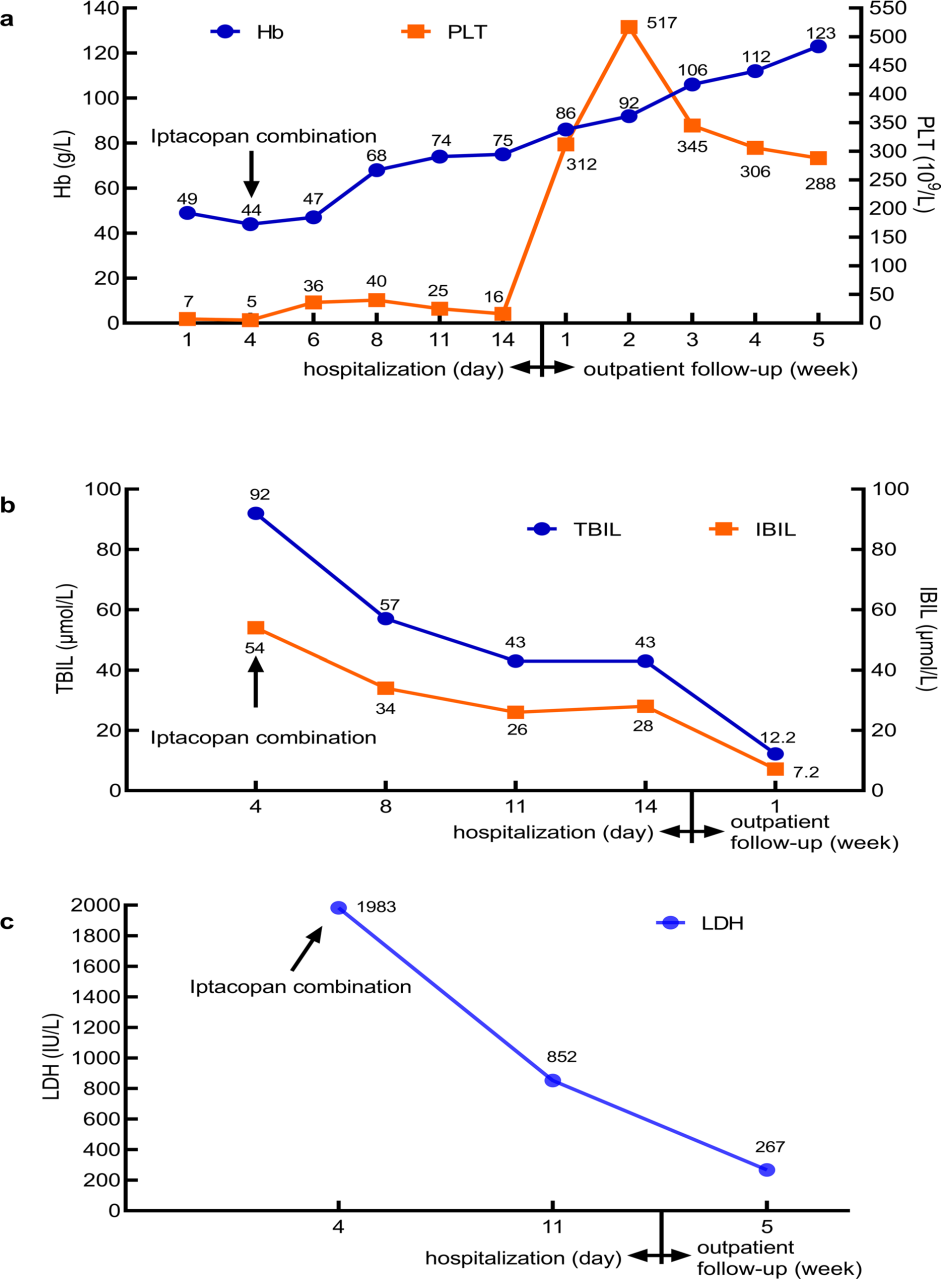
The x-axis represents time points: values ≤ 14 indicate hospitalisation days, while values > 14 correspond to outpatient follow-up weeks. (**a**) Hemoglobin (Hb; reference range: 130-175g/L) and platelet count (PLT; reference range: 125-350 × 109/L). (**b**) Total bilirubin (TBIL; reference range: 3-22 μmol/L) and indirect bilirubin (IBIL; reference range: 0-19 μmol/L). (**c**) Lactate dehydrogenase (LDH; reference range: 120-246 IU/L)

Following transfer to the hematology ward, the patient received methylprednisolone (120 mg once daily) for three days. Laboratory results at day 4 revealed Hb 44 g/L, PLT 5 × 10^9^/L (Fig. 1a), reticulocyte percentage 26.09%, absolute reticulocyte count 330 × 10^9^/L, TBIL 92 µmol/L, IBIL 54 µmol/L (Fig. 1b), LDH 1983 IU/L (Fig. 1c), and creatinine 144 µmol/L. Despite 11 days of high-dose methylprednisolone (120 mg once daily) and repeated plasma exchange, the disease progressed rapidly with renal impairment, a sharp decline in platelet count, severe anemia, and no improvement in hemolytic markers. The patient remains in a state of acute severe microangiopathic hemolysis. Given the delayed efficacy of rituximab, an additional drug was urgently needed to rapidly improve the condition. We have again discussed the patient’s condition with his family. To clarify the causes for poor therapeutic outcomes of previous therapies and identify more effective treatment options, the measurement of the terminal complement complex (C5b-9) and complement factor Ba (Ba) is necessary to confirm activation of the alternative complement pathway in the disease pathogenesis. Elevated levels of terminal complement complex (C5b-9, 302 ng/ml) and complement factor Ba (1800 ng/ml) indicate the activation of the alternative complement pathway during disease progression. Consequently, the complement factor B inhibitor iptacopan (200 mg twice daily) was initiated on day 4 alongside plasma exchange (3000 mL). From day 5, high-dose methylprednisolone (500 mg daily) was administered for 5 days, continuing iptacopan (200 mg twice daily). Additionally, the patient received rituximab on day 5 (100 mg), day 6 (500 mg and 100 mg sequentially). Transient agitation was controlled with sedation. The patient was housed in a laminar flow room during treatment and had a single episode of low-grade fever (37.6ºC) during antibiotic therapy in the hematology ward. From day 1 to day 6, the patient received a total of 800 mL of plasma and 4 units of red blood cells, after which transfusion independence was achieved. By day 8, laboratory tests showed significant improvement: Hb rose to 68 g/L, and PLT rose to 40 × 10^9^/L (Fig. 1a). Iptacopan (200 mg twice daily) was maintained. Methylprednisolone was tapered to 250 mg daily (day 10), then to 120 mg daily (day 15), and 60 mg daily (day 18). The patient’s status improved from critical to severe by day 14, with three sequential doses of rituximab (100 mg, 100 mg, 500 mg). After rituximab administration on day 14, the absolute B lymphocyte (CD19+) count was 0/µL.

By day 5 of iptacopan combination therapy, the patient demonstrated significant hematological improvement, with Hb rising from 44 g/L to 68 g/L and PLT increasing from 5 × 10^9^/L to 40 × 10^9^/L (Fig. 1a). By day 9 of iptacopan therapy, the patient exhibited marked neurological improvement, with restored alertness, resolution of agitation, and reduced fatigue. Cutaneous ecchymoses on both upper and lower limbs also showed substantial regression. By day 11 of iptacopan therapy, Hb reached 75 g/L (Fig. 1a) and serum creatinine normalized. By day 14 of iptacopan therapy, subsequent ADAMTS13 testing revealed severe deficiency (< 0.2 IU/dL) but the absence of inhibitory antibodies (anti-ADAMTS13 IgG negative). This case represents relapsed and refractory iTTP with rapid progression and critical illness. Initial treatment with plasma exchange and corticosteroids resulted in a suboptimal response, whereas adjunctive therapy with iptacopan and rituximab prompted rapid clinical and laboratory improvement. This regimen may serve as a reference for managing relapsed and refractory iTTP cases, particularly in patients with aggressive disease progression.

## Follow up

After discharge, the patient’s general condition was confirmed to be satisfactory, and the patient has completed vaccination against encapsulated bacteria as required. Following discharge, the patient was maintained on oral iptacopan combined with prednisolone. Three days post-discharge, the patient developed pyrexia (peak temperature 39.5°C), which resolved with intravenous immunoglobulin and antipyretic therapy. Serial laboratory monitoring demonstrated progressive hematological improvement: at the 1-week post-discharge review, Hb 86 g/L, PLT 312 × 10^9^/L (Fig. 1a), with normal total and indirect bilirubin levels. By week 2, Hb had risen to 92 g/L and PLT to 517 × 10^9^/L (Fig. 1a), accompanied by significant symptomatic improvement. Continued hematological recovery was observed: week 3 (Hb 106 g/L, PLT 345 × 10^9^/L (Fig. 1a), ADAMTS13 activity increased to 16.2 IU/dL); week 4 (Hb 112 g/L, PLT 302 × 10^9^/L (Fig. 1a), occasional schistocytes, two large ecchymoses on left forearm and right lower limb; and week 5 (Hb 123 g/L, PLT 288 × 10^9^/L (Fig. 1a), LDH 267 IU/L (Fig. 1c), with normalised ADAMTS13 activity). Throughout this period, the corticosteroid dosage was progressively tapered.

## Discussion

The pathogenesis of TTP stems from deficient activity of ADAMTS13, the von Willebrand factor-cleaving protease. Deficient ADAMTS13 activity leads to impaired degradation of ultra-large VWF multimers (UL-VWF) abnormally released from endothelial cells. These UL-VWF multimers spontaneously bind platelets, triggering microvascular thrombosis and microangiopathic hemolysis [1]. Secreted megadimeric VWF and endothelial cell-anchored megadimeric VWF serve as highly adhesive sites capable of initiating platelet adhesion and aggregation, while also activating the alternative complement pathway [9, 12]. Ultra-large Von Willebrand factor multimers anchored on the endothelial cell surface may promote the assembly of AP C3 convertases, thus enhancing complement activation on endothelium, endothelial damage, and intravascular thrombosis [13]. In vitro, C3b (the activated form of C3) binds endothelial-anchored UL-VWF multimers, nucleating formation of both C3 convertase (C3b-Bb) and C5 convertase (C3b-Bb-C3b) complexes on these multimers [12].

A 2018 study analysed plasma levels of C4d, Bb, iC3b, and sC5b-9 in 73 patients with iTTP and healthy controls. The study found no significant increase in C4d levels in acute iTTP patients. However, plasma levels of Bb, iC3b, and sC5b-9 were significantly elevated compared to healthy controls. Notably, Bb levels correlated positively with creatinine and LDH, while sC5b-9 correlated with LDH. These findings suggest that excessive activation of the alternative complement pathway may contribute to acute iTTP pathophysiology [10]. A 2023 study involving 58 acute iTTP patients demonstrated that sC5b-9 levels positively correlated with creatinine and LDH, but inversely correlated with platelet count. The results indicate that complement activation frequently occurs during the acute phase of iTTP, with higher activation levels observed in patients with renal impairment [9]. These studies suggest that complement inhibitors hold therapeutic potential for iTTP. However, there are currently no reported clinical applications of complement inhibitors in iTTP patients.

Iptacopan is a highly potent, reversible, and selective small-molecule inhibitor of factor B. The serine protease factor B (FB) is a key node in the alternative pathway and is integral to the formation of C3 and C5 convertase. Through upstream blockade of complement activation and amplification, iptacopan suppresses factor B to inhibit C3 convertase activity. This mechanism reduces C3 generation and accumulation, thereby preventing AP-dependent C3 activation and membrane attack complex (MAC) formation. Consequently, iptacopan mitigates both terminal C5-mediated intravascular hemolysis and proximal C3-mediated extravascular hemolysis [14, 15].

Standard of care treatment has evolved to include TPE, corticosteroids, and rituximab [4]. However, refractory disease and relapse risk remain concerns [6]. Caplacizumab is a humanized monoclonal antibody that binds to vWF, blocking its interaction with platelet glycoprotein Ib-IX-V and reducing microthrombi formation. Caplacizumab received its first global approval on 3 September 2018 in the EU [16]. However, the incorporation of caplacizumab into clinical practice remains controversial and variable. One concern is its high cost (a treatment course, according to drug labeling, costs 270 000 USD) and cost-ineffectiveness according to 1 analysis [17, 18]. Several real-world observational studies, mostly based on routinely collected data, have reported that the use of caplacizumab in conjunction with TPE and immunosuppression (e.g., corticosteroids and rituximab), known as the triple therapy, has significantly accelerated the disease recovery, shortened the hospital stay, reduced the number of TPE sessions, and reduced exacerbations and mortality rates [19–22].

The treatment landscape for iTTP has been revolutionized by the introduction of caplacizumab. The combination of therapeutic plasma exchange with corticosteroids, rituximab, and caplacizumab has significantly reduced mortality and morbidity in iTTP. This drug has now become part of the therapy in conjunction with plasma exchange and immunosuppression in international guidelines [20]. However, the use of this agent was not feasible during the treatment period of the patient in this study. Caplacizumab has not yet been approved for marketing in China by the National Medical Products Administration (NMPA). Due to the lack of formal approval, it is not routinely available for clinical use in China and may only be accessible through specific clinical trials—subject to eligibility and ethical approval—but remains unavailable to general patients through standard medical channels. Consequently, the standard first-line treatment regimen in most Chinese medical centers, including ours, remains based on plasma exchange, corticosteroids, and rituximab. This highlights significant socioeconomic and geographical disparities in access to novel therapies.

In contrast, iptacopan, used in our case, offers a novel mechanism by inhibiting alternative complement pathway activation, targeting a more specific event in the pathophysiology of endothelial injury and inflammation. This mechanistic difference suggests that complement inhibition might provide a novel pathway for TTP patients. Furthermore, a theoretical potential for synergy exists: caplacizumab prevents platelet aggregation while iptacopan mitigates complement-mediated endothelial assault. Future studies exploring therapies would be of interest.

Notably, prior literature contains no reports of complement inhibitors being used to treat TTP. In this case, the patient’s second TTP episode was more severe and progressed more rapidly than the initial presentation. During acute, severe microangiopathic hemolytic anemia with biochemical evidence of alternative pathway activation, prompt in-hospital treatment over 18 days followed by 5 weeks of outpatient monitoring yielded significant clinical improvement. The patient regained full consciousness, exhibited marked fatigue reduction, achieved normalisation of hemoglobin, platelet counts, body temperature, and renal function, and became transfusion-independent with substantially improved quality of life. The favorable outcome in this case of relapsed/refractory TTP was observed following a combination regimen that included iptacopan alongside high-dose glucocorticoids, plasma exchange, and rituximab.

The results of an observational study indicate that the complex therapy of an acute TTP episode (PEX series and immunosuppressive treatment) results in the disappearance of ADAMTS13 inhibitors and also in the decrease of complement activation [23]. The observed clinical remission in this case report was temporally associated with the administration of iptacopan; however, this does not automatically establish a direct causal relationship. The comprehensive improvement in the patient’s clinical symptoms and laboratory parameters was likely the result of the combined effects of plasma exchange, high-dose glucocorticoids, rituximab, and iptacopan. The therapeutic response in this case may have stemmed from a synergistic effect. The combined use of a complement inhibitor for rapid control of the disease crisis, on the foundation of conventional immunosuppressive therapy, may have jointly contributed to the observed rapid and stable clinical remission. This treatment strategy provides a complete practical example. This observation warrants validation in future larger prospective cohort studies or randomized controlled trials to clarify the potential beneficial role of complement inhibitors.

Our report has several important limitations. First and foremost, this is a single case report without a control group. Therefore, the observed clinical improvement cannot be definitively attributed to iptacopan, as it may have been influenced by the concurrent immunosuppressive therapy or other factors. Secondly, the evidence level is low, and our primary aim is to generate a hypothesis regarding the potential utility of complement inhibition in refractory TTP. The promising role of iptacopan and other complement inhibitors in this setting must be rigorously evaluated in prospective clinical trials before any clinical recommendations can be made. A further limitation of our study is the non-use of caplacizumab, reflecting the current accessibility challenges in our healthcare setting. This may affect the generalizability of our conclusions to regions where this drug is integrated into standard first-line therapy.

The patient was an adult with a sporadic onset, and ADAMTS13 autoantibodies were detected on multiple occasions; therefore, genetic testing was not necessary. ADAMTS13 genetic testing is generally reserved for patients with persistent severe ADAMTS13 deficiency without detectable inhibitors, early childhood onset, or family history [7].

## Data Availability

No datasets were generated or analysed during the current study.

## Abbreviations

TTP: thrombotic thrombocytopenic purpura
cTTP: congenital thrombotic thrombocytopenic purpura
iTTP: immune thrombotic thrombocytopenic purpura
MAHA: microangiopathic hemolytic anemia
TPE: therapeutic plasma exchange
PLT: platelet count
LDH: lactate dehydrogenase
VWF: Von Willebrand factor
MAX: membrane attack complex
AP: alternative pathway
TBIL: total bilirubin
IBIL: indirect bilirubin
Hb: hemoglobin
EICU: emergency intensive care unit
NMPA: National Medical Products Administration
